# A rapid and sensitive bioassay for the simultaneous measurement of multiple bone morphogenetic proteins. Identification and quantification of BMP4, BMP6 and BMP9 in bovine and human serum

**DOI:** 10.1186/1471-2121-10-20

**Published:** 2009-03-19

**Authors:** Blanca Herrera, Gareth J Inman

**Affiliations:** 1 Growth Factor Signalling Laboratory, The Beatson Institute for Cancer Research, Garscube Estate, Switchback Road, Bearsden, Glasgow, G61 1BD, UK

## Abstract

**Background:**

Bone morphogenetic proteins (BMPs) are pleiotropic members of the TGF-beta superfamily which regulate many biological processes during development and adult tissue homeostasis and are implicated in the pathogenesis of a number of human diseases. Their involvement in both normal and aberrant physiology creates a need for rapid, sensitive and methodologically simple assays to evaluate their activity from a variety of biological samples. Previously alkaline phosphatase based assays, ELISA and luciferase based bioassays have been developed to evaluate either individual or total BMP activity. In this paper, we describe a highly sensitive, rapid and specific cell based assay for the simultaneous quantification of total and isoform specific BMP activity from biological samples.

**Results:**

A C2C12 cell line stably transfected with a reporter plasmid consisting of the BMP response element (BRE) from the Id1 promoter fused to a luciferase reporter gene was generated. Exposure of this cell line to human recombinant BMP2, BMP4, BMP6, BMP7, BMP9 and BMP10 induced the expression of luciferase which was quantified using a luminometer. This assay was specific for BMP activity as the other TGF-β superfamily members TGF-β 1, Nodal and Mullerian Inhibiting Substance (MIS) did not induce the reporter. Pretreatment of samples with isoform specific BMP blocking antibodies coupled with isoform specific titration analysis allowed the simultaneous identification and quantification of BMP4, BMP6 and BMP9 in serum samples.

**Conclusion:**

The assay is rapid (<48 hours) and can be used to simultaneously measure isoform specific and total BMP activity in complex solutions.

## Background

Bone morphogenetic proteins (BMPs) are members of the TGF-β superfamily and were originally identified by their ability to induce endochondral bone formation [1]. There are at least 20 BMP family members and they are involved in a myriad of biological processes both during embryonic development and adult life. These include pluripotency of embryonic stem cells [2], dorsoventral patterning of the mesoderm, neurogenesis, hematopoiesis, somite formation, osteoblastic differentiation and bone homeostasis [3-5].

BMPs are synthesized as precursor proteins that are intracellularly proteolytically cleaved following dimerization to produce active mature protein dimers. The BMPs signal via hetero-oligomeric complexes of combinations of three type II receptors:- BMP receptor type II (BMPR2), activin A receptor type IIa (ACVR2A/ACTRII) and ACVR2B (ACTRIIB) and four type I receptors:- ACVRL1 (Activin like kinase 1, ALK1), ACVR1 (ALK2), BMPR1A (ALK3) and BMPR1B (ALK6) [6-9]. Following ligand induced receptor hetero-oligomerization the type I receptors are activated by type II receptor mediated phosphorylation events. The receptor complexes then transduce their signals via activation of the canonical Smad pathway and several non-Smad signalling pathways. The receptor regulated Smads (R-Smads), Smad1, Smad5 and Smad8 are directly phosphorylated by the type I receptor kinases which enables complex formation with the co-Smad, Smad4. R-Smad/Smad4 complexes accumulate in the nucleus and regulate target gene expression by binding to gene regulatory elements and recruiting transcriptional co-repressor and/or activation complexes [10].

As well as playing critical roles in normal physiological processes, dysregulation of BMP signalling can have pathophysiological consequences [11]. Mutations of BMP receptors have been observed in several human pathologies. For example, inactivation of ALK1 results in Hereditary Hemorrhagic Telangiectasia type 2 (HHT2) [12] and mutations of BMPR2 are found in primary pulmonary arterial hypertension patients [13] and pancreatic cancers [14]. Similarly, polymorphisms/mutations have also been observed in BMPs in several human pathologies [11]. Evidence is also accumulating to suggest that aberrant expression of the BMPs may also have pathological consequences. Decreased expression of BMP7 in primary breast cancer specimens has been associated with bone metastasis [15] and is reduced in advanced prostate adenocarcinoma [16]. In contrast elevated BMP7 levels correlate with shorter tumour recurrence in malignant melanoma [17] and increased BMP7 levels in colorectal cancer correlates with depth of tumour invasion, liver metastasis, advanced Duke's classification and poor prognosis [18]. Elevated levels of BMP7 have also been observed in the synovial fluid from rheumatoid arthritis patients [19]. BMP4 levels have also been found to increase in late colonic adenocarcinomas and are higher in primary colonic carcinomas with liver metastasis than matched normal mucosa [20]. BMP6 levels have been observed to decrease in diffuse large B cell lymphoma and correlate with reduced survival [21] and to be elevated in prostate cancer [22]. BMP2 expression has also been observed to be elevated in lung tumours [23].

The observations that BMP signalling may play important roles in both normal and aberrant physiology suggest that the ability to conveniently measure BMP bioactivity in biological samples may have clinical diagnostic and prognostic value and this has driven the development of ELISA [24,25], enzyme linked immunoreceptor assays (ELIRA) [26] and cell based assays to measure BMP activity [27]. The inhibitor of differentiation transcription factor Id1 is an immediate early BMP target gene and the BMP responsive elements of the human and mouse Id1 promoters have been well characterised [28]. Synthetic engineering of two copies of the BMP response elements of the mouse Id1 gene enabled the generation of a highly specific and sensitive BMP responsive luciferase based reporter construct termed BRE-Luc [28]. Recent reports have described the development of BRE-Luc based bioassays in stable reporter cell lines which are capable of measuring the activity of BMP2, 4, 6 and 7 [29,30]. Here we describe the development of a C2C12 BRE-Luc bioassay cell line. We demonstrate that this cell line is capable of measuring the activity of BMP2, BMP4, BMP6, BMP7, BMP9 and BMP10 at physiological concentrations and show that by using isoform specific blocking antibodies this assay can be used to measure simultaneously the levels of BMP4, BMP6 and BMP9 in fetal calf serum (FCS) and human serum samples. This assay paves the way for measuring the activity of multiple BMPs in complex biological samples.

## Results

### Generation of a stable BMP reporter cell line

The BMP responsive C2C12 mouse myoblast cell line was stably transfected with the BRE-Luc construct which contains the BMP responsive elements of the mouse Id1 gene cloned into the pGL3 luciferase vector [28]. Multiple stable clones were tested for BMP inducibility in media containing 0.1% serum by treatment with 5 ng/ml recombinant BMP9 (GDF2). Clone 22 (named C2C12BRE) was found to be the most sensitive (data not shown) and was used for all subsequent experiments. This cell line was maintained in 0.7 mg/ml G418 for routine passage and tested at each passage for BMP responsiveness. We found no change in the BMP responsiveness of C2C12BRE cells measured over 20 passages and after multiple rounds of storage in liquid nitrogen and re-culture (data not shown).

### Dose dependent induction of luciferase by multiple BMPs in C2C12BRE cells

**Figure 1:**
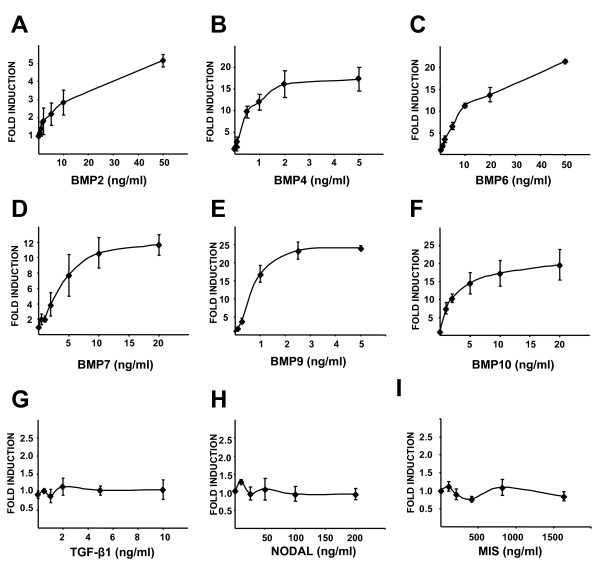
**BMP-specific dose dependent induction of BRE-Luc activity in C2C12BRE cells**. C2C12BRE cells were seeded overnight in 24 well plates at 2 × 10^4 ^cells per well and serum starved in DMEM containing 0.1% FCS for 7 hours. After serum starvation, cells were incubated without or with increasing concentrations of recombinant human (A) BMP2, (B) BMP4, (C) BMP6, (D) BMP7, (E) BMP9, (F) BMP10, (G) TGF-β1, (H) Nodal and (I) MIS. After 16 hours of treatment, BRE-luciferase activity was assessed by measuring luciferase activity in cell lysates. Luciferase activities were normalised to protein content and fold inductions relative to untreated samples were determined. Each point represents the mean ± SEM of three independent experiments performed in quadruplicate.

**Figure 2:**
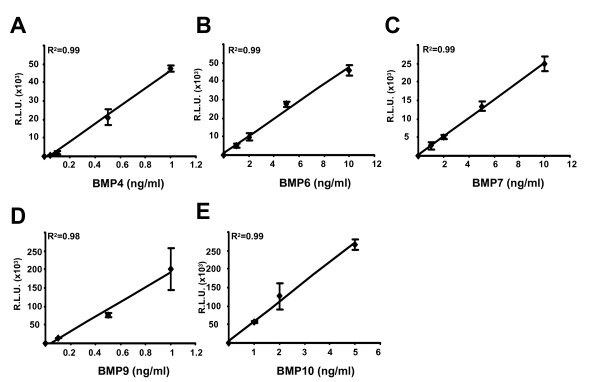
**Determination of the linear range of activation of BRE-Luc by BMPs**. C2C12BRE cells were treated as in Figure 1 with increasing concentrations of (A) BMP4, (B) BMP6, (C) BMP7, (D) BMP9 and (E) BMP10. Luciferase activity was normalised to protein content. Relative luciferase units (RLU) following background subtraction are shown. Data shown is a representative experiment performed in quadruplicate ± SD.

Having established that the C2C12BRE cells were sensitive to BMP9 treatment we investigated the dose dependent effects of multiple recombinant BMPs on this cell line. Cells were incubated with increasing concentrations of recombinant human BMP2, BMP4, BMP6, BMP7, BMP9 and BMP10. All 6 BMPs tested stimulated luciferase activity in a dose dependent manner, with maximal stimulation reached with >50 ng/ml (>2 nM) BMP2, 2 ng/ml (77 pM) BMP4, >50 ng/ml (>1.66 nM) BMP6, 10 ng/ml (427 pM) BMP7, 5 ng/ml (205 pM) BMP9 and 20 ng/ml (820 pM) BMP10 (Figure 1A–F and data not shown). We next assessed the specificity of the assay for BMPs by treating the cells with other members of the TGF-β superfamily. TGF-β1, Nodal and Mullerian Inhibiting Substance (MIS) failed to increase luciferase activity, even at concentrations as high as 10 ng/ml (400 pM) TGF-β1, 200 ng/ml (7.75 nM) Nodal or 1640 ng/ml (70 nM) MIS (Figure 1G–I). Having observed that BMPs stimulate luciferase production in C2C12BRE cells over a range of concentrations in an isoform specific fashion, we next determined the linear range of the assay with each BMP tested. We found that the assay maintained linearity up to 1 ng/ml (40 pM) BMP4, 10 ng/ml (333 pM) BMP6, 10 ng/ml (427 pM) BMP7, 1 ng/ml (40 pM) BMP9 and 5 ng/ml (205 pM) BMP10 (Figure 2A–E and data not shown). Using these titration analyses we also determined the lower sensitivity limits for each BMP. We could reproducibly measure as little as 0.05 ng/ml (2 pM) BMP4, 0.5 ng/ml (16.5 pM) BMP6, 1 ng/ml (42 pM) BMP7, 0.1 ng/ml (4.1 pM) BMP9 and 1 ng/ml (41 pM) BMP10 (Figure 2 and data not shown). Thus, this assay is sensitive and robust enough to measure 0.05–1 ng/ml (2–40 pM) BMP4, 0.1–1 ng/ml (4–40 pM) BMP9, 0.5–10 ng/ml (16.5–333 pM) BMP6, 1–10 ng/ml (42–427 pM) BMP7 and 1–5 ng/ml (40–205 pM) BMP10.

### Simultaneous measurement of multiple BMPs from biological samples

**Figure 3:**
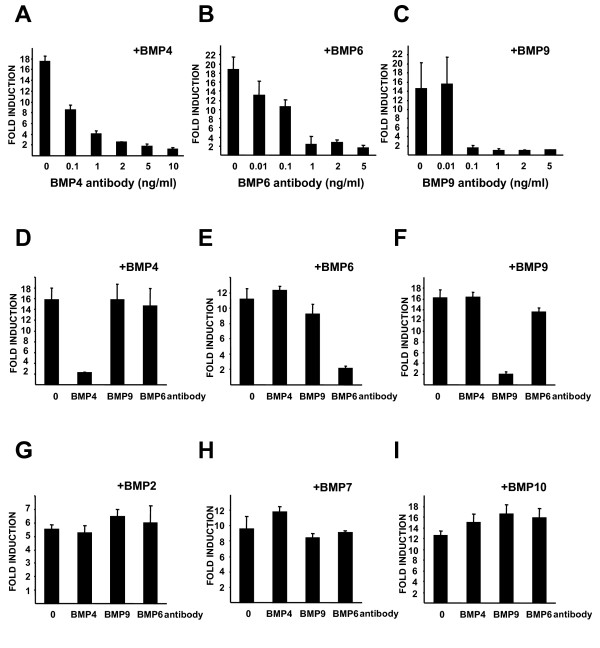
**Inhibition of BMP mediated activation of BRE-Luc with specific blocking antibodies**. C2C12BRE cells were treated as in Figure 1 with (A) 5 ng/ml BMP4, (B) 20 ng/ml BMP6 and (C) 5 ng/ml BMP9 with or without increasing concentrations of BMP4, BMP6 and BMP9 specific blocking antibodies respectively (D, E, F, G, H, I). C2C12BRE cells were treated as in Figure 1 with 5 ng/ml BMP4 (D), 20 ng/ml BMP6 (E), 2 ng/ml BMP9 (F), 50 ng/ml BMP2 (G), 10 ng/ml BMP7 (H) or 20 ng/ml BMP10 (I) in the presence or absence of 5 ng/ml BMP4 antibody, 2 ng/ml BMP6 antibody or 0.1 ng/ml BMP9 antibody. Luciferase activity was normalised to protein content and fold inductions relative to non-BMP treated samples are shown. Each point represents the mean ± SEM of three independent experiments performed in quadruplicate.

The ability of the assay to measure many different BMPs over physiological concentrations prompted us to investigate if we could use the assay in combination with specific blocking antibodies to measure the activity of different BMPs in biological samples. As the assay could measure BMP4, BMP6 and BMP9 over at least a tenfold range of concentrations we focused our efforts on these isoforms. First we determined the ability of commercially available blocking antibodies to block induction of reporter activity by recombinant BMPs. Cells were treated with an excess of recombinant BMPs (5 ng/ml (192 pM) BMP4, 20 ng/ml (666 pM) BMP6 and 5 ng/ml (205 pM) BMP9) with or without a 20 minute pre-treatment with increasing concentrations of their matched blocking antibodies. The minimum concentration required for complete inhibition of BMP induced luciferase activity for each BMP was determined as 5 ng/ml BMP4 antibody (Figure 3A), 2 ng/ml BMP6 antibody (Figure 3B) and 0.1 ng/ml BMP9 antibody (Figure 3C). We next assessed the specificity of these blocking antibodies by treating C2C12BRE cells with several BMPs (BMP2, BMP4, BMP6, BMP7, BMP9 and BMP10) and the blocking antibodies for BMP4, BMP6 and BMP9 (Figures 3D–I). BMP4 induced luciferase activity was only blocked by BMP4 antibody (Figure 3D), BMP6 induced luciferase activity was only blocked by BMP6 antibody (Figure 3E) and BMP9 induced luciferase activity was only blocked by BMP9 antibody (Figure 3F). Likewise, the blocking antibodies only blocked their respective BMP (Figure 3D–I).

**Figure 4:**
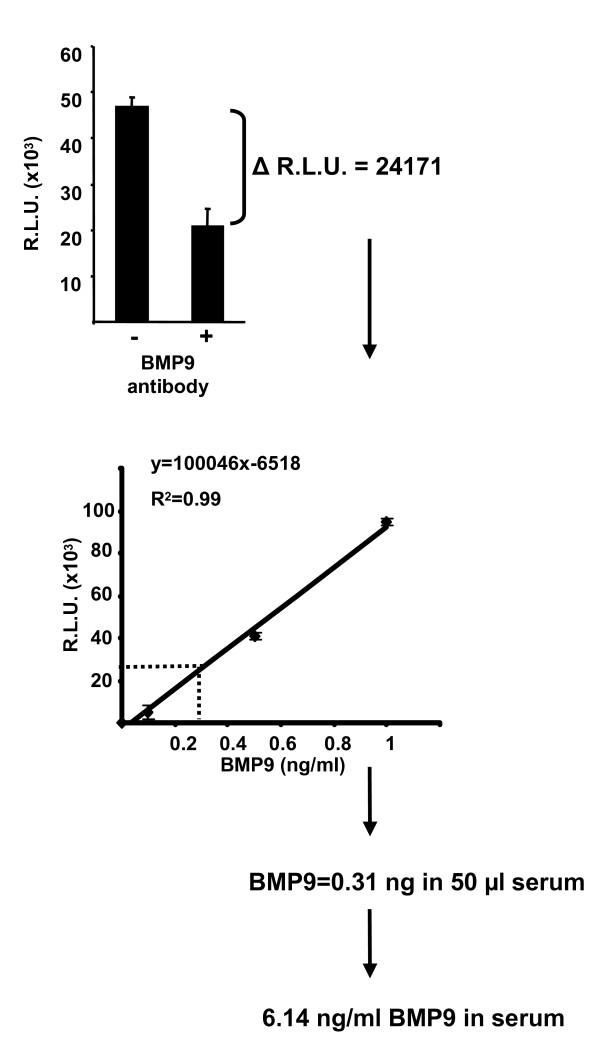
**Methodology for the analysis of BMP9 content inserum**. C2C12BRE cells were treated as indicated in Figure 1 and were stimulated with 10% FCS in the absence or presence of 0.1 ng/ml BMP9 antibody. Luciferase activities were normalised to protein content and relative luciferase units (RLU) were calculated following subtraction of background values obtained from unstimulated cells. The mean decrease of RLU in the presence of BMP9 antibody was calculated (Δ luciferase units). In the same experiment C2C12BRE cells were treated with increasing concentrations of BMP9 within the linear range in order to calculate a standard curve for BMP9 activity as described in Figure 2. The equation of the line was solved and the relative amount of BMP9 calculated using the Δ luciferase value. To obtain total BMP9 concentration per ml of serum this value is multiplied by the dilution factor of 20. Data shown is a representative experiment performed in quadruplicate ± SD.

We next determined that culture of our cells in 10% FCS did not significantly affect cell proliferation or morphology in our assay conditions (data not shown). This fact coupled with the sensitivity of the assay and the availability of the specific blocking antibodies allowed us to develop a method for the quantification of a specific BMP in FCS which we used as a test complex biological sample (See Figure 4). C2C12BRE cells were treated with media containing 10% FCS with and without pre-incubation of the media with 0.1 ng/ml BMP9 blocking antibody. The antibody treated samples produced less relative luciferase units than the untreated samples indicating the presence of BMP9 in this serum sample (Figure 4). C2C12BRE cells were also treated with three different concentrations of BMP9 over the pre-established linear range in parallel to generate a standard curve. By measuring the change in luciferase activity (Δ luciferase) and then extrapolating this value to the BMP9 standard curve we determined that this sample contained 6.14 ng/ml BMP9 (Figure 4).

**Figure 5:**
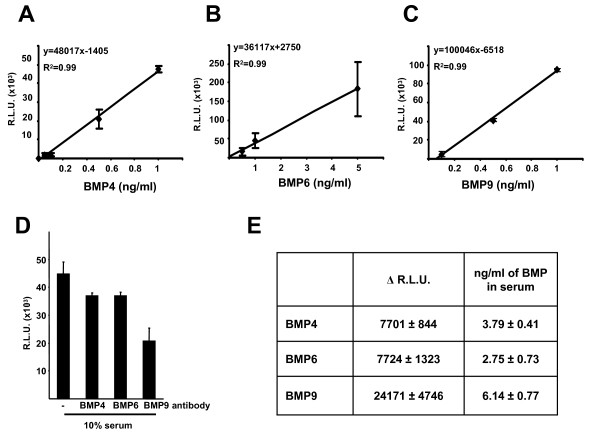
**Simultaneous analysis of multiple BMPs in a serumsample**. C2C12BRE cells were treated as in Figure 4 with increasing concentrations of BMP4 (A), BMP6 (B) or BMP9 (C) within the linear range. Standard curves for (A) BMP4, (B) BMP6 and (C) BMP9 were calculated. (D) Other C2C12BRE cells were incubated with 10% FCS, from Autogen Bioclear, batch 212-181107, in the absence or presence of 5 ng/ml BMP4 antibody, 2 ng/ml BMP6 antibody or 0.1 ng/ml BMP9 antibody. After 16 hours of treatment, cells were lysed and luciferase activity and protein content were determined and the relative luciferase units were calculated as described in Figure 2. (E) Δ luciferase units for each BMP were calculated as described in Figure 4, and the BMP content was calculated by extrapolating the Δ luciferase units' value for each BMP in the appropriate standard curve.

Next we performed a similar analysis by treating cells with three different concentrations within the linear range of recombinant BMP4, BMP6 and BMP9 to generate standard curves (Figure 5A, B, C respectively) and in parallel incubated samples with 10% FCS containing media with and without blocking antibodies to BMP4, BMP6 and BMP9. Treatment with each of the blocking antibodies revealed that FCS contained all three BMPs (Figure 5D). Measurement of Δ luciferase units and extrapolation to the standard curves indicated that BMP4, BMP6 and BMP9 were present in FCS at concentrations ranging from 2.75–6.14 ng/ml (Figure 5E).

**Figure 6:**
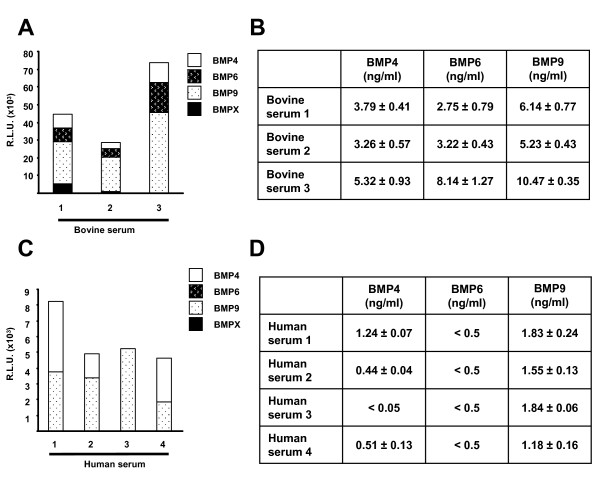
**Analysis of  concentration of multiple BMPs in bovine and human serum samples**. (A and C) C2C12-BRE cells were treated as described in Figure 4 with three separate batches of FCS (bovine serum 1–3) or four different batches of human serum (human serum 1–4) with and without 5 ng/ml BMP4 antibody, 2 ng/ml BMP6 antibody and 0.1 ng/ml BMP9 antibody in separate wells. After 16 hours of treatment, cells were lysed and luciferase activity and protein content were determined and the relative luciferase units were calculated as described in Figure 2. Δ luciferase units for each BMP was calculated as described in Figure 4 and are displayed as part of the total luciferase activity obtained when serum was added in absence of blocking antibodies. Remaining luciferase activity induced by serum but not blocked by BMP4, BMP6 and BMP9 antibodies is labelled as BMPX. (B and D) By extrapolating the Δ luciferase units for each BMP in the appropriate standard curve, the amount of BMP4, BMP6 and BMP9 in each serum was calculated. Data shown is the mean ± SD of a representative experiment performed in quadruplicate.

Having determined that FCS contains BMPs 4, 6 and 9 we tested if the concentrations of these BMPs varied over different batches of FBS obtained from different sources. We found that treatment of the three serum samples with the BMP4, BMP6 and BMP9 blocking antibodies reduced luciferase measurements in all three cases (Figure 6A) indicating that these different batches of serum all contained BMP4, BMP6 and BMP9. Addition of the Δ luciferase obtained with each antibody also indicated that serum 1 and serum 2 samples contained an additional activity capable of inducing the BRE-Luc construct indicating that FCS is likely to contain at least one other BMP (labelled as BMPX, Figure 6A). The total amount of luciferase activity induced by serum stimulation varied approximately two fold between serum samples (Figure 6A) as did the amount of each of the BMPs 4, 6 and 9 (Figure 6B). BMP9 was found in a higher concentration than BMP4 and BMP6 in all samples whereas the relative amounts of BMP4 to BMP6 varied between samples (Figure 6B).

Having identified and quantified BMP4, 6 and 9 in FCS we performed similar analysis on human serum samples. We readily detected BMP4 in three out of four human serum samples in concentrations ranging from 0.44–1.24 ng/ml (Figure 6C, D). Similarly we detected from 1.18 to 1.84 ng/ml BMP9 in all four human serum samples tested (Figure 6C, D). We did not detect BMP6 or any other BMP activity in human serum samples (Figure 6C). Taken together these findings indicate that our assay is capable of determining the relative amounts of BMP4, BMP6 and BMP9 from complex biological solutions.

## Discussion

The pleiotropic and fundamental roles of BMPs in both normal physiological and pathophysiological processes, indicates that the ability to measure BMP activity may have clinical use. In this paper we describe the development of a highly sensitive and specific assay to measure BMP activity from biological samples. We generated a stable cell clone "C2C12BRE" expressing a luciferase reporter under the control of the BMP response elements from the mouse Id1 promoter. When compared to other available bioassays, our assay has enhanced BMP isoform range, increased sensitivity and is capable of simultaneously measuring multiple individual BMP isoforms from the same complex biological samples.

The most widely used cell based assay to assess BMP activity employs measurement of alkaline phosphatase activity in C2C12 myoblast cells [27]. However, this assay may lack sufficient sensitivity to measure physiological levels of BMPs, takes several days to perform and is susceptible to influence from other signalling pathways [31,32]. Recently, other cell based assays using C3HT10T1/2 embryonic mouse cells [30], C2C12 and HepG2 cells [29] have been described. These assays are capable of measuring BMP2, BMP4, BMP6 and BMP7. We show that our assay is also capable of measuring BMP9 levels from 4–200 pM and BMP10 levels from 40–205 pM. Our assay has enhanced sensitivity for BMP4 (2 pM compared to 3 pM), BMP6 (16.5 pM compared to 40 pM) and BMP7 (42 pM compared to 125 pM). ELISA assays are commercially available for the measurement of BMP4, BMP6 and BMP7 but are currently unavailable for BMP9 and BMP10. BMP4 ELISAs are reported to measure 0.03–2 ng/ml (RnD Systems, USA) and 0.015–1 ng/ml (Raybiotech, USA) similar to the sensitivity of our assay (0.05–1 ng/ml). An ELISA for BMP6 is reported to measure 0.08–8 ng/ml (Raybiotech, USA) similar to our assay sensitivity range (0.5–10 ng/ml). A BMP7 ELISA is reported to have enhanced sensitivity but limited linearity (0.01–1 ng/ml, Raybiotech, USA) when compared to our bioassay (1–10 ng/ml). Our assay therefore has similar sensitivity to ELISA assays but has the advantage that it measures biologically active BMP and not total BMP levels. Importantly, we develop the bioassay methodology further and show that by using BMP isoform specific blocking antibodies it is possible to measure the specific activity of individual BMPs in complex biological samples whereas previous methods have so far only been capable of detecting total BMP activity. Furthermore using recombinant BMPs and isoform specific blocking antibodies we demonstrate that is possible to simultaneously quantitate total BMP activity and individual isoform BMP activity from the same biological sample.

As shown in Figure 1 our assay is specific for BMPs as related TGF-β superfamily members TGF-β, Nodal and MIS had no effect on luciferase activity. By careful titration analysis we were able to define the linear ranges of activity for different BMP isoforms. Our assay is most sensitive for measuring the activity of BMP4, BMP6 and BMP9 and is capable of measuring as little as 40 pM of BMP7 and BMP10 (Figure 2). As we could measure BMP4, BMP6 and BMP9 over greater than 10 fold ranges of concentrations coupled with the availability of isoform specific blocking antibodies for these species we focused our efforts on developing the assay using these BMPs. Using recombinant BMPs and blocking antibodies we demonstrated that it is possible to determine the specific activity of individual BMPs (Figure 3).

We next used FCS as a test complex biological fluid for determining the versatility of our assay. By performing titration analysis with recombinant BMP9 and serum stimulation of C2C12BRE cells with and without BMP9 blocking antibody we discovered that FCS contains high levels of biologically active BMP9 ranging from 5–10 ng/ml (Figures 4, 5, 6). Whilst this paper was in preparation, Sabine Bailly and colleagues discovered that BMP9 is present in human serum at similar concentrations from 2–12 ng/ml and acts as a circulating vascular quiescence factor [33]. Our data indicates that BMP9 may also play roles during bovine development and reveals a remarkably conserved steady state level of BMP9 in serum samples between species. BMP9 has been shown to signal via ALK1 in endothelial cells [8] and indicates that either C2C12 cells express functional ALK1 or that BMP9 may also engage alternative receptors. We are currently investigating these hypotheses. Our analysis also revealed that FCS contains BMP4 ranging from 3–5 ng/ml and BMP6 ranging from 2.75–8 ng/ml (Figure 6). BMP4 has previously been purified from FCS [34] but our study is the first to measure the concentration of BMP4 in FCS. To our knowledge this current study is the first to identify BMP6 in FCS. Our experiments also revealed that in two out of three FCS samples the combined activity of BMP4, BMP6 and BMP9 could not account for the total BMP activity in these samples. We therefore conclude that FCS may also contain at least one other BMP isoform (BMPX, Figure 6). Remarkably, our studies reveal that FCS contains high levels of BMP activity and that typical cell culture conditions of 5–10% FCS contain BMP activity from 0.5–2 ng/ml of BMPs. Given the profound biological effects of BMP signalling on cell biology and the interaction of these events with other signalling cascades, we believe that it is important to consider the potential effects of BMP signalling when assessing tissue culture experimental results.

As well as measuring BMPs in FCS we expanded our analysis and found that BMP9 is present in human serum in 1–2 ng/ml quantities in concordance with recently published findings [33]. To the best of our knowledge we identify BMP4 as a human serum factor for the first time. We failed to detect BMP6 or any other BMP activity capable of inducing BRE-Luc in human serum indicating that BMP4 and BMP9 maybe the only circulating BMPs present in normal human serum.

An increasing body of evidence is revealing that aberrant BMP signalling and changes in BMP levels may have important biological consequences during normal and pathophysiological processes [11]. We have demonstrated that our bioassay is capable of measuring the activity of individual BMP isoforms from serum samples. It is an exciting possibility that determination of BMP levels in patient biological samples such as serum, or body cavity fluids may have prognostic and/or diagnostic utility in the management of human disease. Recent studies have also suggested the possibility of using recombinant BMPs to treat osteogenic disease [35]. The use of BMP bioassays may also be useful in monitoring the bioactivity of BMPs in these clinical settings. Our assay could also be used in determining the specificity and efficacy of agents designed to target BMP signalling. With the increasing availability of commercial BMP blocking antibodies and recombinant proteins it should be possible to broaden the applicability of our bioassay. Furthermore, by extrapolating the methodology we describe new cell lines capable of measuring further isoforms of BMP for the use in laboratory research and clinical practice could be developed.

## Conclusion

We have generated a rapid, sensitive and specific bioassay for the simultaneous measurement of total and individual isoform BMP activity from complex biological solutions. This assay can be used to study BMP activity in experimental and clinical settings and to screen for pharmacological modifiers of BMP signalling.

## Methods

### Reagents

Recombinant human TGF-β was purchased from Peprotech (Peprotech, UK). Recombinant human BMP2, BMP4, BMP6, BMP7, BMP9, BMP10, MIS and Nodal were purchased from R&D Systems Inc. Each growth factor was resuspended in 1 mM HCl/1 mg/ml BSA and used at appropriate concentrations. The following blocking antibodies were purchased from R&D Systems and resuspended in PBS and used at the appropriate concentrations: monoclonal anti human BMP9 antibody (MAB3209), polyclonal anti human BMP6 antibody (AF507) and monoclonal anti human BMP4 antibody (MAB757). Different batches of FCS were tested for the presence of BMPs. Batch 108005 from Autogen Bioclear (bovine serum #1), batch 212-181107 from Autogen Bioclear (bovine serum #2) and batch CSE0442 from Perbio (bovine serum #3). Different batches of human serum were purchased from Sigma, 078K1708 (human serum #1), 078K1707 (human serum #2), 078K1823 (human serum #3) and 117k1692 (human serum #4).

### Generation of reporter cell line

C2C12 mouse myoblast cell line was cultured in Dulbecco's modified Eagle's medium (DMEM) supplemented with 2 mM L-glutamine, 100 U/ml of penicillin, 100 U/ml streptomycin and 10% fetal calf serum (FCS) and were grown at 37°C in 10% CO_2_. To generate BMP reporter cell line, C2C12 cells were stably transfected with pGL3(BRE)-luciferase reporter construct [28]. 0.6 × 10^6 ^C2C12 cells were plated in a 10 cm dish and were transfected the next day with 8 μg pGL3(BRE)-luciferase reporter construct and 1 μg G418 resistant plasmid pTK-neo [36] using Hiperfect transfection reagent (Qiagen, Crawley, UK) according to manufacturer's instructions. Three days later the cells were plated at different densities and selected for antibiotic resistance using 700 μg/ml G418. Individual clones were isolated, expanded and tested for BMP inducibility of luciferase expression. Clone 22 was selected and grown in the presence of 700 μg/ml G418, named C2C12-BRE and used for all subsequent experiments.

### BMP bioassay

C2C12-BRE cells were plated at a concentration of 2 × 10^4 ^per well in 24 well plates containing DMEM plus 10% FCS and allowed to attach for 18 hours. Cells were washed with PBS and re-fed with 0.5 mls of DMEM 0.1% FCS for 7 hours. Recombinant growth factors were added to cells at the appropriate concentrations for 15 hours and then cells were washed with PBS and lysed using 100 μl of reporter lysis buffer (Promega, Madison, USA). To measure luciferase activity, 40 μl of lysate was added to 40 μl Luciferase Assay Reagent (Promega) and luminescence was quantitated using a Veritas Microplate Luminometer. The protein concentration of each lysate was analyzed using Bio-Rad protein assay reagent according to the manufacturer's instructions (Biorad, USA). Luciferase units obtained were normalized to the protein content of each well. All experiments were performed at least three times with four independent wells per condition.

### BMP bioassay for the analysis of serum samples

Standard curves for each BMP to be analyzed were generated as above with BMP concentrations spanning the linear range of each BMP in each experiment. 100 μl serum samples were divided in half and were treated or untreated for 20 minutes with specific BMP blocking antibodies at the concentration determined to be required to block maximal recombinant BMP activation. Samples were then diluted to 500 μl in DMEM and incubated with the C2C12-BRE cells exactly as described above. Luciferase activity was measured and normalised to protein content and background values obtained from cells grown in DMEM 0.1% FCS alone were subtracted to generate relative luciferase units (RLU). RLU values obtained in the presence of the blocking antibody were subtracted from the RLU values obtained without blocking antibody to generate a Δ luciferase unit value. This value was used to calculate the amount of each BMP assayed in the serum samples by extrapolation from the standard curves.

## Authors' contributions

BH and GJI performed and designed experiments and drafted the manuscript. All authors read and approved the manuscript.
